# Complete mitochondrial genome and the phylogenetic position of the common cuckoo, *Cuculus canorus bakeri* (Aves: Cuculiformes)

**DOI:** 10.1080/23802359.2019.1660256

**Published:** 2019-09-02

**Authors:** Shi Qiu, Hua Liu, Yan-Sen Cai, Wei Hou, Liang Dou, Xiu-Yue Zhang, Jing Li

**Affiliations:** Key Laboratory of Bioresources and Ecoenvironment (Ministry of Education), College of Life Sciences, Sichuan University, Chengdu, China

**Keywords:** *Cuculus canorus bakeri*, complete mitochondrial genome, phylogenetic analysis

## Abstract

The complete mitochondrial genome of Common Cuckoo (*Cuculus canorus bakeri*) is determined in this study and was deposited in Genbank with accession number MN067867. The length of the mitogenome is 17,457 base pairs (bp) including 13 protein-coding genes (PCGs), 22 transfer (t RNA) RNA genes, two ribosomal RNA (r RNA) genes, one control region (CR) and one short pseudo-control region. The structure is similar to mitochondrial genome of other Cuculiforme species. Phylogenetic tree shows that *C. canorus bakeri* is closely related to *C. poliocephalus*. The study has provided useful information for further studies on the evolution, genetic diversity, and phylogenetic relationships in Common Cuckoo.

The parasitic cuckoos (Cuculiformes, Cuculidae) account for two-thirds of all obligate brood parasitic birds (Davies 2000). As one of parasitic cuckoos, many previous research related to *Cuculus canorus* revolved around its calls (Wei et al. 2015) or its relationship with host species (Stoddard and Stevens 2010). *Cuculus canorus bakeri,* the subspecies of *C. canorus,* is mainly distributed in the northeast Indian subcontinent, Assam, Nepal, Bhutan, northern Southeast Asia, southeastern Tibet and southern China (Mann 2013). *Cuculus canorus bakeri* pronounces the advertising call ‘cu-coo’ like other subspecies of *C. canorus* (Lei et al. 2005). To date, only four complete mitochondrial genomes have been reported in the Cuculiforme, which limits the better understanding of these parasitic birds. This study presents the complete mitochondrial genome of *C. canorus bakeri* to enrich the genetic information on the parasitic bird.

The muscle sample from a natural died individual of *C. canorus bakeri* was collected from Laojunshan National Nature Reserve, Yibin, Sichuan Province, China (104°00.99′, 28°41.98′). The specimen was stored in the Natural Museum of Sichuan University with a voucher number of 2019052101. In total, 32 primer pairs were designed to acquire the whole mitochondrial genome of Common Cuckoo by polymerase chain reaction. The length of complete mitogenome is 17,457 bp, containing two r RNA genes (12Sr RNA and 16Sr RNA), 13 protein-coding genes, 22 t RNA genes, one control region and one short pseudo-control region. The architecture of mitochondrial genome of Common Cuckoo is similar to that of other Cuculiforme species. The genome sequence has been deposited in the Genbank with the accession number MN067867. The base composition is 33.08% for A, 24.24% for T, 29.78% for C and 12.9% for G. The content of A + T is 57.31%, which is similar to the mitochondrial genome of greater Coucal (Qu et al. 2017).

To further understand relationship of *C. canorus bakeri* to other Cuculiformes species, DNA sequences of 13 protein-coding genes in *C. canorus bakeri*, four Cuculiformes species and *Caprimulgus indicus* were used for phylogenetic analysis. Three methods (MP/ML/NJ) were applied to constructed the phylogenetic tree (Figure 1). According to the tree, *C. canorus bakeri* has a closer relationship to *C. poliocephalus* than to other species. The result is consistent with previous study (Wang et al. 2017). The sequence data will benefit study on the evolution of *C. canorus bakeri* and the co-evolutionary pattern between *C. canorus bakeri* and its hosts.

**Figure 1.**
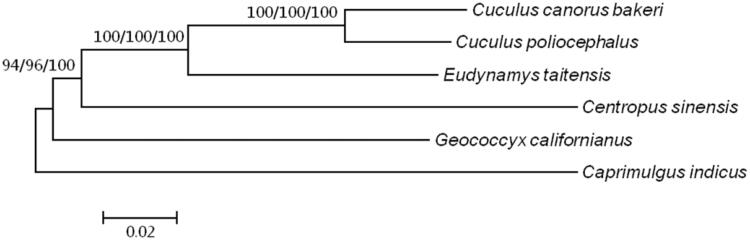
Estimation of phylogeny using 13 concatenated mitochondrial PCGs for six species. Three methods were used to construct the phylogenetic tree, maximum-parsimony method, maximum-likelihood method and neighbour-joining method, which constructed by PAUP4.0, PAUP4.0 and MEGA5.2 individually. Sequence data used in the study are the following: *Cuculus canorus bakeri* (MN_067867), *Cuculus poliocephalus* (NC_028414), *Eudynamys taitensis* (NC_011709), *Centropus sinensis* (KT947122), *Geococcyx californianus* (NC_011711), *Caprimulgus indicus* (NC_025773). *Caprimulgus indicus* species was set as outgroup.
